# Insulin resistance-related features are associated with cognitive decline: a cross-sectional study in adult patients with type 1 diabetes

**DOI:** 10.1186/s13098-023-01249-w

**Published:** 2024-01-11

**Authors:** Xiaolin Ji, Wenjing Zou, Li Fan, Zhiguang Zhou, Xiongzhao Zhu, Xia Li

**Affiliations:** 1 National Clinical Research Center for Metabolic Diseases, Key Laboratory of Diabetes Immunology (Central South University), Ministry of Education, Department of Metabolism and Endocrinology, The Second Xiangya Hospital of Central South University, 410011 Changsha, Hunan China; 2 National Clinical Research Center for Mental Disorders, Medical Psychological Center, Medical Psychological Institute of Central South University, The Second Xiangya Hospital of Central South University, 410011 Changsha, Hunan China; 3 Department of Radiology, The First Affiliated Hospital of Zhejiang University School of Medicine, 310000 Hangzhou, China

**Keywords:** Type 1 Diabetes, Cognitive function, Insulin resistance, Estimated insulin sensitivity

## Abstract

**Background:**

To investigate the associations between insulin resistance (IR)-related features and cognitive function in type 1 diabetes (T1D).

**Methods:**

A total of 117 adult patients with T1D were recruited in this cross-sectional study. IR-related features include overweight/obesity/central obesity, hypertension, atherogenic dyslipidemia, and decreased estimated insulin sensitivity (eIS). The Wechsler Memory Scale-Chinese Revision, Wisconsin Card Sorting Test, and Sustained Attention to Response Task was used to assess memory, executive function and sustained attention, respectively. A z-score was generated from each test, and a composite measure of global cognitive performance was calculated by averaging the z-scores of all tests. Cognitive differences were measured between T1D patients with and without IR-related features. The associations between IR-related features and and cognitive performance were analyzed using: logistic regression, partial correlation, and multivariate linear regression analysis.

**Results:**

A total of 53 (45.3%) T1D patients were defined as having IR-related features. Individuals with IR-related features displayed worse overall cognitive scores compared to those without and had a 4-fold increase in the risk for having global cognitive z-score < 0. Among the IR-related features, higher triglyceride (TG) and lower eIS showed linear correlation with lower global cognitive performance. And the subsequent regression analysis identified eIS as the factor independently associated with global cognitive performance.

**Conclusions:**

We have provided evidence linking IR-related features to deteriorated cognitive function in adult patients with T1D. And eIS showed an independent positive correlation with global cognitive performance. Although no causal relationship can be drawn, IR emerges as an important factor reflecting cognitive function.

**Trial registration:**

ClinicalTrials.gov NCT03610984.

**Supplementary Information:**

The online version contains supplementary material available at 10.1186/s13098-023-01249-w.

## Background

Type 1 diabetes (T1D) is a chronic disease characterized by persistent autoimmune destruction of β cells, leading to lifelong dependence on exogenous insulin [1]. The incidence rate of T1D is increasing annually by 2–3% worldwide [2]. According to the latest report from the diabetes atlas of the International Diabetes Federation (IDF), T1D patients have reached 8.75 million, and 5.56 million (64.0%) of them are between 20 and 59 years old [3]. Patients with T1D have a higher risk of developing a series of chronic complications, affecting prognosis and quality-of-life [4].

In addition to classical diabetic complications, diabetes-associated cognitive dysfunction has attracted increasing attention in recent years, which mainly manifests as deficits in executive functioning, learning and memory, attention, and visual-spatial ability [5]. These cognitive domains play important roles in problem-solving and self-control, and T1D-related cognitive deficits have been found related to decreased self-management behaviors and worse glycemic control [6]. Research on risk factors for cognitive dysfunction finds that a longer duration of diabetes, earlier disease onset, poorer glycemic control and the presence of diabetic complications contribute to but do not fully explain the increased risk of cognitive deficits in T1D [7]. Therefore, the pathogenesis of T1D-related cognitive deficits remains incompletely understood and requires further investigation.

Autoimmune destruction of pancreatic β cells is central in the pathogenesis of T1D, yet insulin resistance (IR) is also commonly present [8]. Since the gold standard method to measure IR, the hyperinsulinemic euglycemic clamp, is time-consuming and difficult to perform [9], indirect measures are often used for IR assessment. Metabolic abnormalities, including obesity, atherogenic dyslipidemia, hypertension, metabolic syndrome as well as specific calculators are often used as clinical surrogates to represent IR [10]. A 3 ~ 50% prevalence of IR in T1D patients has been reported based on these surrogate assessments, which is higher than that in the general population [11] and can well predict the risk of chronic diabetic complications [8].

The brain is identified as an insulin-sensitive organ, and IR is proposed as the link of type 2 diabetes (T2D) to cognitive decline [12], whereas the association between IR and cognitive deficits has rarely been discussed in T1D. By analyzing the data set from Diabetes Control and Complications Trial/ Epidemiology of Diabetes Interventions and Complications study (DCCT/ EDIC) study, cognitive decline occurring in T1D was found associated with estimated glucose disposal rate (eGDR), a specific calculator to measure IR in T1D population [13, 14]. However, it remains unclear if other IR-related metabolic abnormalities are associated with cognitive decline in T1D. Therefore, the aim of the current study was to examine the associations between factors representing IR and cognitive function within T1D adult group.

## Methods

### Participants

A total of 117 participants with T1D were recruited through the Diabetes Outpatient Clinic at the Second Xiangya Hospital. The present data were collected from January 2016 to March 2022. Detailed inclusion and exclusion criteria are as follows. Inclusion criteria: (1) diabetes diagnosed according to the World Health Organization criteria of 1999 [15]; (2) clinically diagnosed as T1D: (a) positive for at least one of the islet autoantibodies, including glutamic acid decarboxylase antibody (GADA), insulinoma-associated 2 molecule antibody (IA-2A), and zinc transporter 8 antibody (ZnT8A); (b) insulin dependency from disease onset; (c) diabetic ketoacidosis (DKA)/ diabetic ketosis (DK) at onset; (d) continuous loss of β-cell function (postprandial C-peptide < 300 pmol/L); (e) onset age ≤ 30 years old and > 6 months old (meet a and b; or negative for all three islet autoantibodies but meet b and any term of c ~ e); 3) age between 18 ~ 60 years old; 4) duration of diabetes ≥ 6 months. Exclusion criteria: (1) other types of diabetes (e.g., monogenic diabetes, disease of the exocrine pancreas, and drug or chemical-induced diabetes); (2) histories of psychiatric or neurological disorders and other chronic or acute diseases; (3) history of long-term alcohol consumption; (4) using statins or other medications affecting lipid profiles.

### Evaluation of clinical and laboratory variables

Demographic data and laboratory parameters were collected, including gender, age, age at onset, duration of diabetes, height, weight, waist circumference (WC), hip circumference, blood pressure (BP), educational levels, history of hypoglycemia and DK/DKA episodes, diabetic complications (diabetic retinopathy, neuropathy, and nephropathy), daily insulin dose, depression levels assessed by the Beck Depression Inventory (BDI), anxiety levels assessed by the State-trait Anxiety Inventory (STAI), glycated hemoglobin (HbA1c), lipid profiles (triglyceride, TG; high-density lipoprotein-cholesterol, HDL-C), fasting C-peptide (FCP), stimulated C-peptide (preserved residual β-cell function refers to FCP or stimulated C-peptide ≥ 16.7 pmol/L), islet autoantibodies (GADA, IA-2A, and ZnT8A).

To account for the degree of chronic exposure to hyperglycemia, a “hyperglycemia exposure score” was calculated [16]. All available HbA1c values of each patient were collected from medical records at the Second Xiangya Hospital. First, diabetes duration and median HbA1c were transformed into z-scores. Second, each patient’s diabetes duration z-score was added to his or her median HbA1c z-score, with higher summed z-score indicating higher degree of hyperglycemia exposure. This summed z-score thus reflects diabetes disease duration and degree of glycemic control, allowing us to distinguish between two patients with similar median HbA1c values but different duration of disease.

### Insulin resistance (IR)-related features

For adult patients, overweight was defined as a 24 < body mass index (BMI) ≤ 28 kg/m^2^ and obesity as a BMI > 28 kg/m^2^. Central obesity was defined as a waist circumference ≥ 90 cm for males and ≥ 85 cm for females regardless of BMI. Hypertension was defined as repeated BP measurements ≥ 130/85 mmHg or confirmed diagnosis of hypertension receiving antihypertensive medications. Atherogenic dyslipidemia refers to either a TG level ≥ 1.70 mmol/L or HDL-C level < 1.04 mmol/L. eGDR = 24.31–12.22 × waist-to-hip ratio (WHR) − 3.29 × (hypertension: 1 if present, 0 if absent) − 0.57 × HbA1c mg/kg/min. A lower eGDR level indicates greater IR [14].

To investigate the impact of IR on cognition independent of the glucose levels, we utilized a calculator for IR that does not include blood glucose indicators. The estimated insulin sensitivity (eIS) is a calculated index previously developed and validated against hyperinsulinemic-euglycemic clamp data from Coronary Artery Calcification in Type 1 Diabetes (CACTI) Study for estimating IR in adults with T1D. eIS = exp (4.1075 − 0.01299 [waist, cm]-1.05819 [insulin dose, daily units per kg]-0.31327 [triglycerides, mmol/L]-0.00802 [DBP, mmHg]) [17]. A lower eIS level indicates greater IR. The cut off point of eIS ≤ 4.66 mg/kg/min was adopted in the current study [18].

The patient was defined as having IR-related features when meeting any of the following criteria: BMI > 24 kg/m^2^, central obesity, hypertension, elevated TG, decreased HDL-C, and eIS ≤ 4.66 mg/kg/min. And the continuous variables to assess IR include BMI, WC, systolic blood pressure (SBP), diastolic blood pressure (DBP), HDL-C, TG and eIS.

### Evaluation of cognitive function

#### Memory

Verbal and visual memory were measured with subtests of the Wechsler Memory Scale-Chinese Revision (WMS‐RC) [19]. For the verbal memory task, the examiner read each of the two stories once, and the patients were asked to recall the stories with as much detail as possible, both immediately and again after a 30‐min delay. In the visual reproduction task, patients were presented with three pictures (10s per picture), and were asked to draw the picture they saw, both immediately and again after a 30‐min delay.

#### Executive function

Executive function was assessed using the Wisconsin Card Sorting Test (WCST) [20], a well-established and widely recognized test used to assess global executive function, including the ability to handle complex problem-solving and maintain mental flexibility in light of changing stimuli. The patients were required to sort stimulus cards based on different matching rules (color, shape, or number), without being informed that the rules of sorting have changed. Each patient was scored for total errors, perseverative errors, non-perseverative errors, and number of completed categories (T-scores), with the lower score indicating more severe executive function damage.

#### Sustained attention

Sustained attention was measured by the Sustained Attention to Response Task (SART) [21], a widely used measurement of attention. Patients were asked to respond to the “Go” target and withhold a response to the “No-go” target. The reaction time (RT) for each “Go” target was recorded. The proportion of omission errors represents failures to respond to the “Go” targets and commission errors represents failures to withhold response to the “No-go” targets. The SART included a total of 225 trials, and intra-individual variability (IIV) represents the trial-to-trial RT fluctuation, measured as RT standard deviation/mean RT.

The three cognitive tests above were administered to participants in fixed order by trained examiners when participants’ blood glucose levels were in the range of 4.0 ~ 17.0 mmol/L. Raw scores were standardized by z-scale using mean and standard deviation of the entire subject pool (n = 117), and an average z-score was calculated for each cognitive domain to capture general performance, with a higher average z-score indicating better performance [22]. And the global cognitive performance was estimated by averaging the z-scores from each of the three cognitive domains (memory, executive function and sustained attention). For each specific cognitive domain and global functioning, subjects were split into “high” and “low” cognitive performance groups based upon a median z-score cut-off of 0.

### Statistical analysis

Data analysis was performed in Statistical Product and Service Solutions (SPSS) software (IBM Statistics for Macintosh, version 26.0; IBM Corporation, Armonk, New York). Continuous variables were expressed as the median (25th-75th percentiles), and categorical variables were presented in the form of the number (%). Shapiro-Wilk test was applied to assess distributions of continuous variables. The intergroup comparisons were performed by Mann-Whitney U test for non-normal distributed continuous variables, independent-samples T test for normally distributed continuous variables, and Chi Square test for categorical variables. Logistic regression analyses with odds ratios (ORs) and 95% confidence intervals (CIs) were performed to assess the associations between IR-related features and cognitive performance. Bivariate correlations between IR-related metabolic parameters and cognitive performance were analyzed using Spearman correlations, and a multiple linear regression model was developed to further test the independent associations (forward selection stepwise; *P* < 0.05 criterion for variable retention). Prior to correlation and regression analysis, variables were Box-Cox transformed to achieve normality. Two-tailed *p*-values < 0.05 were considered significant. Data analysis steps are summarized in Supplementary Fig. 1.

## Results

### General characteristics of T1D patients

This study included 117 T1D patients, and their median age was 26.6 (21.1, 32.7) years, the median diabetes duration was 3.3 (1.6, 8.6) years, the proportion of male participants was 41.0%, and the proportion of autoimmune T1D (with at least one positive islet autoantibodies) was 76.9%.

Overall, 11 (9.4%) participants had overweight/obesity/central obesity, 14 (12.0%) had hypertension, 21 (17.9%) had abnormal TG or HDL-C, 38 (32.5%) had eIS ≤ 4.66 mg/kg/min. Taken together, 53 (45.3%) participants had IR-related features, with 31 (26.5%) having 1 IR-related feature and 22 (18.8%) having ≥ 2 IR-related features. The detailed characteristics are presented in Table 1.

**Table 1:** Characteristics of the participants

Characteristics	T1D (n = 117)
Male (n, %)	48 (41.0%)
Age (years)	26.6 (21.1, 32.7)
Age at onset (years)	21.0 (14.7, 28.5)
Duration of diabetes (years)	3.3 (1.6, 8.6)
GADA+, IA-2A+, or ZnT8A+ (n, %)	90 (76.9%)
Insulin pump users (n, %)	83 (70.9%)
Daily insulin dose (u/kg/day)	0.62 (0.46, 0.74)
Preserved residual β-cell function (n, %)	66 (56.4%)
HbA1c (%)	7.4 (6.5, 8.6)
Diabetic complications (n, %)	11 (9.4%)
eGDR (mg/kg/min)	9.9 (8.8, 10.7)
Overweight/obesity/central obesity (n, %)	11 (9.4%)
Hypertension (n, %)	14 (12.0%)
Atherogenic dyslipidemia (n, %)	21 (17.9%)
eIS ≤ 4.66 mg/kg/min (n, %)	38 (32.5%)
Having IR-related features (n, %)	53 (45.3%)

Data are expressed as number (%) or median (25th-75th percentiles). GADA: glutamic acid decarboxylase antibody; IA-2A: insulinoma-associated protein 2 antibody; ZnT8A: zinc transporter 8 antibody; HbA1c: glycated hemoglobin; eIS: estimated insulin sensitivity; IR: insulin resistance

### Cognitive difference between T1D patients with and without IR-related features

Compared to T1D patients with no IR-related features, patients with IR-related features had worse global cognitive performance [-0.11 (-0.73, 0.16) vs. 0.15 (-0.27, 0.45), *p* = 0.008] (i.e., the average performance of memory, executive function and sustained attention). Among the three cognitive domains, patients with IR-related features performed worse on tests of executive function, showing significantly worse general performance [-0.16 (-0.76, 0.29) vs. 0.18 (-0.34,0.66), *p* = 0.025], lower scores of preservative errors [45.0 (37.5, 49.0) vs. 47.0 (40.5, 54.0), *p* = 0.036] and fewer completed categories [3.0 (2.5, 4.0) vs. 4.0 (3.0, 4.0), *p* = 0.027]. Although the general performance of memory and sustained attention showed no difference, patients with IR-related features performed worse on immediate visual memory task [10.0 (8.0, 11.5) vs. 11.0 (9.0, 12.0), *p* = 0.034], made more commission errors [0.48 (0.32, 0.68) vs. 0.28 (0.16, 0.56), *p* = 0.026] and had higher intra-individual variability [0.26 (0.20, 0.32) vs. 0.23 (0.18, 0.28), *p* = 0.021] (Table 2).

**Table 2:** Characteristics of the participants with and without IR-related features

Characteristics	With no IR-related features (n = 64)	With IR-related features (n = 53)	*P value*
Male (n, %)	26 (40.6%)	22 (41.5%)	1.000
Age (years)	27.5 (23.6, 33.1)	23.2 (19.6, 32.3)	0.087
Age at onset (years)	23.6 (18.1, 29.5)	17.6 (13.1, 25.4)	**0.010**
Duration of diabetes (years)	2.8 (1.4, 6.2)	5.3 (1.9, 10.0)	0.076
Preserved residual β-cell function (n, %)	36 (56.2%)	28 (52.8%)	0.855
HbA1c (%)	7.0 (6.3, 8.3)	7.6 (6.6, 9.3)	**0.041**
Hyperglycemia exposure score	-0.65 (-1.15, 0.41)	0.32 (-0.47, 1.25)	**0.001**
Daily insulin dose (u/kg/day)	0.54 (0.44, 0.66)	0.70 (0.52, 0.83)	**< 0.001**
History of DK/DKA (n, %)	49 (76.6%)	50 (94.3%)	**0.017**
Diabetic complications (n, %)	1 (1.6%)	10 (18.9%)	**0.002**
Hypoglycemia episodes of the previous month	2.0 (1.0, 5.0)	1.0 (0.0, 3.0)	**0.004**
Education (years)	15.0 (12.0, 16.0)	15.0 (12.0, 16.0)	0.115
BDI	5.0 (3.0, 12.0)	5.0 (2.0, 9.0)	0.257
STAI-S	36.0 (30.0, 43.0)	38.0 (29.5, 43.0)	0.567
STAI-T	35.0 (31.0, 43.0)	37.0 (32.0, 42.0)	0.919
Global cognitive performance	0.15 (-0.27, 0.45)	-0.11 (-0.73, 0.16)	**0.008**
WMS-RC			
General memory	0.17 (-0.43, 0.57)	-0.12 (-0.71, 0.46)	0.070
Verbal memory-immediate	7.5 (6.1, 8.6)	7.0 (5.0, 8.9)	0.349
Verbal memory-delayed	6.0 (4.2, 8.0)	5.5 (3.4, 7.0)	0.099
Visual memory-immediate	11.0 (9.0, 12.0)	10.0 (8.0, 11.5)	**0.034**
Visual memory-delayed	10.0 (8.0, 11.0)	9.0 (8.0, 11.0)	0.392
WCST parameters			
General executive function	0.18 (-0.34, 0.66)	-0.16 (-0.76, 0.29)	**0.025**
Total errors	49.0 (44.0, 53.0)	47.0 (40.0, 50.5)	0.054
Non-perseverative errors	47.0 (43.5, 55.0)	46.0 (41.0, 49.5)	0.108
Preservative errors	47.0 (40.5, 54.0)	45.0 (37.5, 49.0)	**0.036**
Completed categories	4.0 (3.0, 4.0)	3.0 (2.5, 4.0)	**0.027**
SART parameters			
General sustained attention	0.21 (-0.40, 0.54)	-0.14 (-0.53, 0.40)	0.064
Omission error	0.01 (0.00, 0.02)	0.01 (0.01, 0.04)	0.205
Commission error	0.28 (0.16, 0.56)	0.48 (0.32, 0.68)	**0.026**
IIV	0.23 (0.18, 0.28)	0.26 (0.20, 0.32)	**0.021**
Reaction time (ms)	360.1 (315.4, 492.7)	359.9 (304.5, 409.5)	0.204

Data are expressed as number (%) or median (25th-75th percentiles). An average z-score was calculated for each domain to capture general performance, with higher average z-score equals better performance. And the global cognitive performance was estimated by averaging the z-scores of all three domains. The *p* values for the comparisons of cognitive scores were adjusted for age, gender, educational level, depression and anxiety state. IR: insulin resistance; DK: diabetic ketosis; DKA: diabetic ketoacidosis; BDI: the Beck Depression Inventory; STAI-S: the State-trait Anxiety Inventory-State; STAI-T: the State-trait Anxiety Inventory-Trait; WMS-RC: the Wechsler Memory Scale-Chinese Revision; WCST: the Wisconsin Card Sorting Test; SART: the Sustained Attention Response Task; IIV: intra-individual variability

Patients with IR-related features also had earlier onset of T1D, reported higher daily insulin dose, exhibited higher hyperglycemia exposure, had a higher prevalence of DK/DKA and diabetic complications, and experienced more hypoglycemia episodes. Meanwhile, there was no difference in terms of age, gender distribution, disease duration, β-cell function, years of education, or the depression and anxiety state (Table 2).

### Associations between IR-related features and cognitive function

To explore the associations between IR-related features and cognitive function, patients were divided into “high” and “low” cognitive performance groups based upon a median z-score cut-off of 0 (Table 3). After adjustment of potential confounders, logistic regression analysis demonstrated that individuals with IR-related features showed a 4-fold increased risk of having low global cognitive performance (adjusted OR = 4.07, 95% CI 1.44–9.62) as compared with subjects with no IR-related features. In addition to the global cognitive function, having IR-related features was also associated with an increased risk of having low memory performance (adjusted OR = 6.03, 95% CI 1.38–26.39), low executive function performance (adjusted OR = 2.74, 95% CI 1.06–7.08) and low sustained attention performance (adjusted OR = 3.78, 95% CI 1.20-11.92).

**Table 3:** ORs and 95%CIs for the associations between IR-related features and cognitive performance

Components	Having low global cognitive performance	Having low memory performance	Having low executive function performance	Having low sustained attention performance
Having IR-related features^†^	4.07 (1.44, 9.62) **	6.03 (1.38, 26.39) *	2.74 (1.06, 7.08) *	3.78 (1.20, 11.92) *
Having 1 IR-related feature^†^	3.45 (1.04, 11.44) *	3.32 (0.64, 17.37)	2.34 (0.79, 6.91)	2.11 (0.54, 8.20)
Having ≥ 2 IR-related features^†^	4.86 (1.36, 17.39) *	13.35 (2.10, 84.94) **	3.30 (1.01, 10.86) *	6.86 (1.66, 28.40) **
Overweight/obesity/central obesity	1.72 (0.39, 7.54)	3.89 (0.53, 28.32)	4.97 (1.07, 23.19) *	1.74 (0.36, 8.33)
Hypertension	1.55 (0.42, 5.71)	1.73 (0.34, 8.84)	0.90 (0.26, 3.16)	0.56 (0.11, 2.75)
Atherogenic dyslipidemia	7.08 (1.96, 25.55) **	5.49 (1.03, 29.33) *	3.52 (1.15, 10.84) *	3.58 (0.97, 13.19)
eIS ≤ 4.66 mg/kg/min	3.57 (1.23, 10.34) *	13.89 (2.48, 77.84) **	1.64 (0.65, 4.14)	3.31 (1.04, 10.48) *

Logistic regression analyses were performed to evaluate the associations between IR-related features and cognitive performance, adjusted for age, gender, age of onset, hyperglycemia exposure score, history of DK/DKA, hypoglycemia episodes of the previous month, diabetic complications, years of education, depression and anxiety scores. ORs: odds ratios; CIs: confidence intervals; IR: insulin resistance; eIS: estimated insulin sensitivity. ^†^: compared to having no IR-related features; *: *p* < 0.05, **: *p* < 0.01, ***: *p* < 0.001

Among the IR-related features, overweight/obesity/central obesity was associated with worse executive function (adjusted OR = 4.97, 95% CI 1.07–23.19). Having atherogenic dyslipidemia was associated with low global cognitive performance (adjusted OR = 7.08, 95% CI 1.96–25.55), low memory (adjusted OR = 5.49, 95% CI 1.03–29.33) and executive function (adjusted OR = 3.52, 95% CI 1.15–10.84). Having lower eIS was associated with low global cognitive performance (adjusted OR = 3.57, 95% CI 1.23–10.34), low memory (adjusted OR = 13.89, 95% CI 2.48–77.84) and low sustained attention (adjusted OR = 3.31, 95% CI 1.04–10.48). Whereas no association was observed between hypertension and cognitive performance.

### Independent associations between IR-related metabolic parameters and cognitive function

The bivariate associations between IR-related metabolic parameters and cognitive function were further examined by partial correlation analyses (Table 4). For obesity components, WC was negatively correlated with executive performance (β=-0.230, *p* = 0.019), while BMI showed no association with any cognitive domain. Dyslipidemia components showed associations with global cognitive performance. Specifically, higher TG was associated with lower global cognitive score (β=-0.283, *p* = 0.003), memory (β=-0.371, *p* = 0.002) and executive score (β=-0.257, *p* = 0.008), and higher HDL-C was associated with higher score of sustained attention (β = 0.302, *p* = 0.007). The calculated eIS was positively correlated with global cognitive score (β = 0.296, *p* = 0.002), memory (β = 0.296, *p* = 0.013) and executive score (β = 0.258, *p* = 0.008). Meanwhile, blood pressure still showed no correlation with any of the cognitive performance.

**Table 4:** Correlations of IR-related metabolic parameters with cognitive performance

Components	Global cognitive performance		General memory		General executive function		General sustained attention	
	β	*P*	β	*P*	β	*P*	β	*P*
Body mass index (kg/cm^2^)	-0.033	0.736	0.060	0.620	-0.070	0.482	-0.053	0.644
Waist circumference (cm)	-0.141	0.150	0.011	0.930	**-0.230**	**0.019**	-0.026	0.823
Systolic blood pressure (mmHg)	-0.062	0.531	0.014	0.912	-0.066	0.512	-0.094	0.413
Diastolic blood pressure (mmHg)	-0.086	0.380	-0.174	0.149	-0.106	0.284	-0.089	0.433
TG (mmol/l)	**-0.283**	**0.003**	**-0.371**	**0.002**	**-0.257**	**0.008**	-0.146	0.202
HDL-C (mmol/l)	0.093	0.344	0.204	0.090	-0.017	0.865	**0.302**	**0.007**
eIS (mg/kg/min)	**0.296**	**0.002**	**0.296**	**0.013**	**0.258**	**0.008**	0.199	0.081

Correlation analyses were performed using Spearman correlation, adjusted for age, gender, age of onset, hyperglycemia exposure score, history of DK/DKA, hypoglycemia episodes of the previous month, diabetic complications, years of education, depression and anxiety scores. An average z-score was calculated for each domain to capture general performance, with higher average z-score equals better performance. And the global cognitive performance was estimated by averaging the z-scores of all three domains. IR: insulin resistance; TG: Triglyceride; HDL-C: high-density lipoprotein-cholesterol; eIS: estimated insulin sensitivity

Subsequently, hierarchical multivariate regression analysis was conducted to identify independent associations. Other than IR-related metabolic parameters, age, gender, age of onset, hyperglycemia exposure score, history of DK/DKA, hypoglycemia episodes of the previous month, diabetic complications, years of education, depression and anxiety scores were also incorporated as possible risk factors. Only age (β = 0.256, *p* = 0.004), years of education (β=-0.265, *p* = 0.002), and eIS (β = 0.257, *p* = 0.004) entered the final model for global cognitive function (R^2^ = 0.212, *p* < 0.001).

## Discussion

To our knowledge, this is the first study aimed to examine associations between commonly used indicators of IR and cognitive function within T1D adult population. In the current study, we found that individuals with IR-related features showed a 4-fold increased risk of having low global cognitive performance. Among the IR-related metabolic parameters, eIS showed an independent positive correlation with global cognitive function.

IR is a characteristic usually linked to T2D, but can also be a feature of patients with T1D and their coexistence is called “double diabetes.” IR often arises due to complex interplay between environmental and inherited factors and progresses chronically. Subcutaneous insulin administration rather than the physiological portal vein delivery, is another additional factor in the development of IR in T1D [23]. The prevalence of IR in T1D varies between 3 ~ 50% depending on study population and diagnostic criteria, and the IR status of our participants was similar to that reported in the Chinese population [11]. The increasing trend of IR in T1D is consistent across all measurements, and double diabetes will possibly become the predominant phenotype in T1D in the next few decades [8]. The commonly used measurements of IR are sensitive predictors for T1D-related major complications and mortality [24].

Cognitive deficits can occur even among newly diagnosed children with T1D, and worsen as the disease progresses [5]. Adults with T1D are at increased risk of developing dementia compared to the general population, and the onset occurs at a relatively younger age [25]. But the risk factors contributing to T1D-related cognitive deficits remain to be elucidated. The widespread distribution of insulin receptors on all cell types in the brain suggests the importance of insulin signaling in the brain function. Most insulin in the cerebrospinal fluid derives from circulating pancreatic insulin and enters the brain primarily via the blood brain barrier (BBB). Studies in animals suggest that systemic IR decreases transport of insulin across the BBB and reduces brain insulin levels, leading to impaired insulin signaling pathway and neurosynaptic functioning [26]. Obesity, atherogenic dyslipidemia, metabolic syndrome and IR are closely related to cognitive impairment and dementia among the general population and patients with T2D or Alzheimer’s disease [27, 28]. In T1D, the association between BMI, hypertension and cognition function has been reported, but the findings are discrepant [29]. A comprehensive assessment of the association between IR and cognitive function in T1D is lacking.

Only one study from DCCT/EDIC dataset has demonstrated that cognitive decline is associated with IR in patients with T1D, where eGDR＜5.6 mg/kg/min is used to diagnose IR [13]. Although our patients had higher eGDR, deteriorated cognitive function was noted in those with IR-related features, indicating that more sensitive predictors of cognitive dysfunction may exist. In the current study, higher WC was independently correlated with lower executive function. Since BMI showed no correlation, our findings suggest that the visceral fat depot may be an important contributor to the decline in executive function in T1D. Consistent with our result, larger volumes of visceral fat quantified using magnetic resonance imaging are associated with lower performance of executive function among general population [30], and increased visceral fat depot may independently affect cerebral white matter prior to detectable cognitive changes [31]. Recently, a mendelian randomization study also identified visceral adipose tissue and BMI-adjusted-waist-hip-ratio (WHR) as risk factors with potential causal effects on genetically predicted general cognition [32]. Other than the obesity parameters, dyslipidemia also showed an independent correlation with deteriorated cognitive function. Findings from general population suggest that even modest, preclinical changes in lipid metabolism may be sufficient to evoke changes to hippocampus structure and function, which is responsible for encoding and recalling memories [33]. And simvastatin and lovastatin are drugs that can potentially be used for the treatment of cognitive deficits [34]. In order to investigate the effect of IR on cognition independent of the glucose levels, rather than eGDR containing HbA1c value, the calculated eIS was adopted to represent IR in our study, and eIS showed an independent positive correlation with global cognitive function. It is surprising that glycemic control related parameters did not enter the final model for cognitive prediction, though long-term follow up studies demonstrate that the progression of cognitive impairment is largely explained by HbA1c [7]. This might be explained by the rather fair glycemic control of our study population. Perspective study is needed to verify the role of individual IR-related metabolic components in T1D-related cognitive decline.

Several limitations should be addressed when interpreting results of the current study. First, indirect measurements were used for IR evaluation. It should be noted that parameters examined in our study are less variable than fasting glucose or insulin concentrations, and are widely used in clinical and research approach, making these measurements comparable and reliable. Second, the disease duration of our participants was short, and further studies with larger sample sizes and longer duration are needed to verify these findings. Third, we lacked information regarding macrovascular complications in the present study, precluding any meaningful discussions on their effects. Lastly, we performed the cross-sectional study at a single center with limited number of patients. Since single point of measurements precludes judgments of causality, further studies are needed to investigate the longitudinal relationship between IR and cognitive decline and whether the results apply in other racial/ethnic groups.

The major strength of this study was that we evaluated the relationship between commonly used indicators of IR and cognitive function within T1D adult group. These findings may provide a novel direction in understanding the mechanism of cognitive impairment in T1D, and facilitate clinical guidance and interventions for patients with IR to minimize T1D-related cognitive decline.

### Electronic supplementary material

Below is the link to the electronic supplementary material.

Supplementary Material 1: Main analysis flowchart: T1D: type 1 diabetes; BMI: body mass index; WC: waist circumference; SBP: systolic blood pressure; DBP: diastolic blood pressure; TG: triglyceride; HDL-C: high-density lipoprotein-cholesterol; eIS: estimated insulin sensitivity; IR: insulin resistance; ORs: odds ratios; CIs: confidence intervals

## Data Availability

The datasets of the current study are available from the corresponding author upon reasonable request.

## Abbreviations

T1D: type 1 diabetes
IR: insulin resistance
eIS: estimated insulin sensitivity
eGDR: estimated glucose disposal rate
DK/DKA: diabetic ketosis/ diabetic ketoacidosis
WC: waist circumference
BDI: Beck Depression Inventory
STAI: State-trait Anxiety Inventory
HbA1c: glycated hemoglobin
TG: triglyceride
HDL-C: high-density lipoprotein-cholesterol
BMI: body mass index
SBP: systolic blood pressure
DBP: diastolic blood pressure
WMS-RC: the Wechsler Memory Scale‐Chinese Revision
WCST: the Wisconsin Card Sorting Test
SART: the Sustained Attention to Response Task
IIV: intra-individual variability
RT: reaction time
ORs: odds ratios
CIs: confidence intervals
