# Supplementary figures and images for: Insulin resistance-related features are associated with cognitive decline: a cross-sectional study in adult patients with type 1 diabetes
Source: Diabetol Metab Syndr. 2024 Jan 11;16:13. doi: 10.1186/s13098-023-01249-w (PMC10782534; doi:10.1186/s13098-023-01249-w)

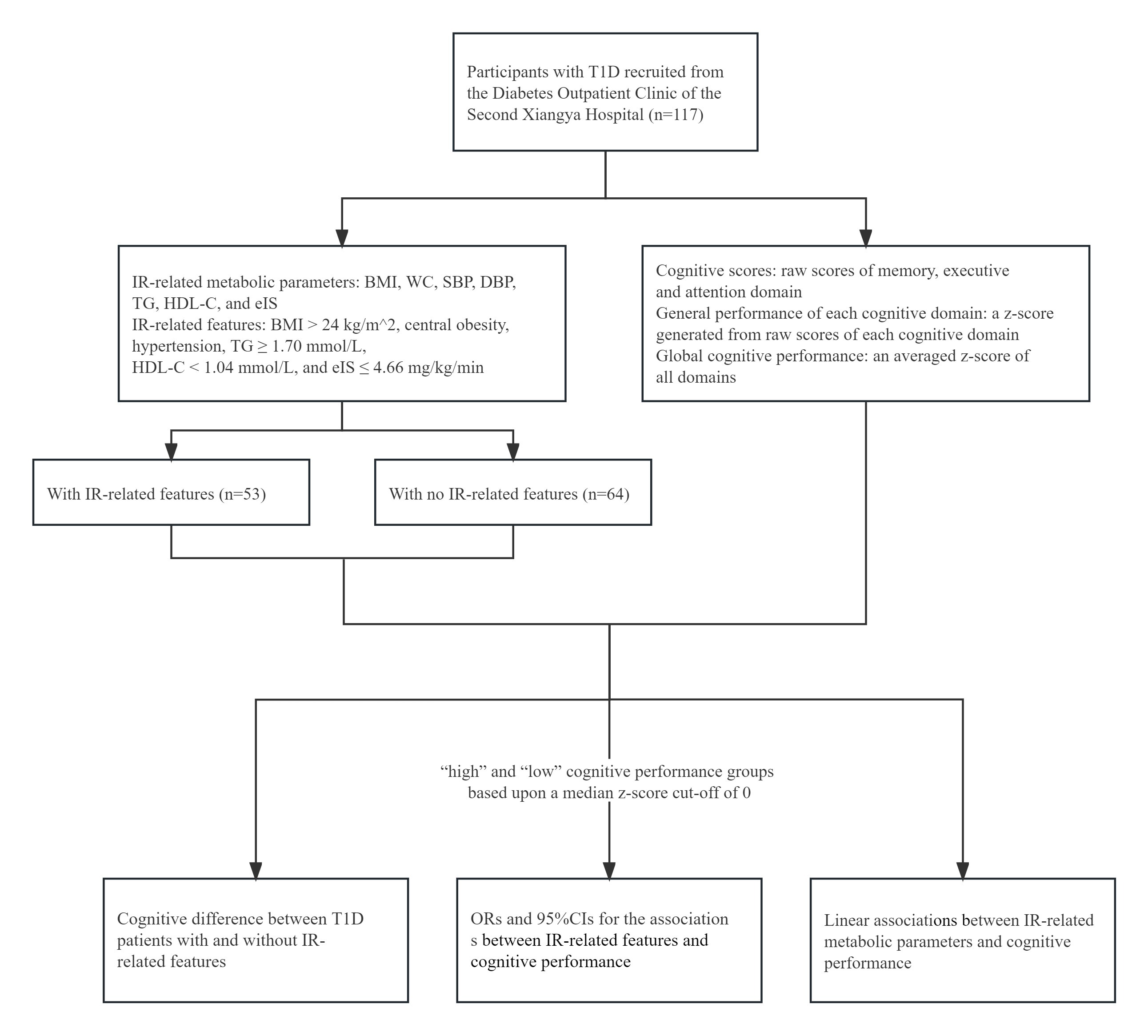

Supplement: Supplementary file 1 — Supplementary Material 1: Main analysis flowchart: T1D: type 1 diabetes; BMI: body mass index; WC: waist circumference; SBP: systolic blood pressure; DBP: diastolic blood pressure; TG: triglyceride; HDL-C: high-density lipoprotein-cholesterol; eIS: estimated insulin sensitivity; IR: insulin resistance; ORs: odds ratios; CIs: confidence intervals [file 13098_2023_1249_MOESM1_ESM.jpg]
